# Prayer for Recovery

**Published:** 1890-07-19

**Authors:** 


### Words of Consolation.

### PRAYER FOR RECOVERY.

Many sick persons are unhappy because they cannot feel certain it is right to pray for their recovery. They are urged by well-meaning but injudicious friends to do so continually, as if they ought to "take Heaven by violence." Others, on the contrary, warn them to take their illness passively, as God knows what is best for them; and by not doing so, they show their want of submission to His will.

But we should not look on our Heavenly Father in the light of a fond and foolish parent, who always gives us what we ask, because we ask it, and who grants all our desires just because he could not deny us anything we set our hearts on. If it were so, we ought certainly never to ask for anything, lest the gift should prove exactly what would harm us. In that case, where would be the comfort or rest in prayer? But God really gives us only those things which are good for us, and denies us those which would prove for our undoing, so we may safely confide all our wishes to Him. We need not fear to tell Him all our hearts with the simple faith and words of a little child at its mother's knee. It is a great proof of our love to God, and of our confidence in His sympathy, when we tell Him all our wants and desires both in bodily and spiritual tribulations, keeping back nothing because it is too trivial for His notice. Has He not told us by His dear Son, that though sparrows are so cheap that two are sold for a farthing, yet not one falls to the ground without our Heavenly Father's knowledge. He loves us to unburthen ourselves thoroughly without concealment, for "He requireth truth in the inward parts."

It is surely the duty of a person at the beginning of an illness to pray that, if it be the will of God, he may recover his bodily health. Even as the sickness increases, and at the latest periods, we may use the words which the Church has provided for one in extremity: "We know, O Lord, that no work is impossible with Thee, and that if Thou wilt, Thou canst even yet raise him up, and grant him a longer continuance amongst us," but it adds, "if it be Thy gracious will." And this seems a fit pattern for all our devotions. Ask for what we want: if it be His will to grant it, it is well; and if not, try to be contented with whatever answer He deigns to send.

We must not shrink from life as some do, as if its cares and duties were greater temptations than those of sickness. We little know ourselves if we suppose this, and have not discovered that it is but a change of temptation. No one can say which are the greater, those of health, or those of sickness, for they are so different, they cannot be compared. God is able to help us under any circumstances whatever, and it would be underrating His power to think otherwise. Of course it is impossible while life remains, ever to know that any one will not recover, and the prayers of the righteous have availed to raise up many from the brink of the grave (whether for weal or for woe God only knows); but when it comes to a life-long sickness, an ailment which will gradually sap away the strength, a thorn in the flesh, as in St. Paul's case, which is not permitted to depart from us, then we must be contented with the assurance, "My grace is sufficient for thee." Doubtless he was content, and only asked day by day for the fulfilment of the promise, and for the power to do and to suffer his Master's will in the state of life unto which it had pleased God to call him. The constant asking to have the burden removed would have, both in his and our cases, only encouraged a restless, dissatisfied spirit; a desire for that which it was not God's will to grant.

Pray then day by day for His help, and for such a measure of His strength and grace as will enable us to do His will. Do not check any inclination to ask for renewed health, leaving the issue in His hands, only beseeching Him to hallow all our crosses in this life, and to crown them hereafter where all tears are wiped away, where there is no more death, neither sorrow nor crying, for all these things have passed away.

